# The syndemic burden of HIV/AIDS in Africa amidst the COVID‐19 pandemic

**DOI:** 10.1002/iid3.544

**Published:** 2021-10-04

**Authors:** Olivier Uwishema, Charles Taylor, Lukman Lawal, Nakyanzi Hamiidah, Isoke Robert, Abdulrasheed Nasir, Elie Chalhoub, Jeffrey Sun, Burak T. Akin, Irem Adanur, Rehema M. Mwazighe, Helen Onyeaka

**Affiliations:** ^1^ Oli Health Magazine Organization Research and Education Kigali Rwanda; ^2^ Clinton Global Initiative University New York New York USA; ^3^ Faculty of Medicine Karadeniz Technical University Trabzon Turkey; ^4^ Faculty of Medicine University of Southampton Southampton UK; ^5^ Department of Medicine, College of Health Sciences University of Ilorin Ilorin Kwara State Nigeria; ^6^ Department of Nursing and Midwifery, Faculty of Health Sciences Lira University Lira Uganda; ^7^ Faculty of Medicine University of Saint Joseph of Beirut Beirut Lebanon; ^8^ Faculty of Health Sciences McMaster University Hamilton Ontario Canada; ^9^ Medical Laboratory Technologist The Mombasa Hospital Mombasa Kenya; ^10^ School of Chemical Engineering University of Birmingham Edgbaston, Birmingham UK

**Keywords:** acquired immune deficiency syndrome, Africa, antiretroviral therapy, coronavirus, COVID‐19, HIV/AIDS, human immunodeficiency virus

## Abstract

**Introduction:** The human immunodeficiency virus/acquired immune deficiency syndrome (HIV/AIDS) has long affected millions of individuals across the globe. Historically, the prevalence of this disease is particularly noted within the African continent. Before the coronavirus disease 2019 (COVID‐19) pandemic, many African countries struggled to effectively manage the increasing burden associated with HIV/AIDS. There is now a need to reassess this in a COVID‐19 pandemic context so that the impact of COVID‐19 on HIV/AIDS healthcare within Africa can be adequately evaluated.

**Methods:** Data collection was performed on the PubMed, Ovid MEDLINE and Embase bibliographical databases with a predefined search strategy. Searches were performed in blind duplicate and all articles considering COVID‐19 and HIV/AIDS within African healthcare were considered.

**Results:** The COVID‐19 pandemic has severely exacerbated the many issues surrounding HIV/AIDS care within many African countries. These impacts are noticeable in medical, psychological, and socio‐political contexts.

**Conclusions:** Before efforts are made to improve the provision of HIV/AIDS and COVID‐19 care within Africa, it is important that this issue is brought to the attention of the scientific and clinical community so that the continent can receive the necessary support and aid.

## 1 INTRODUCTION

Coronavirus disease 2019 (COVID‐19) is a respiratory illness caused by severe acute respiratory syndrome coronavirus 2 (SARS‐CoV‐2).^1^ Coronavirus was first identified in Wuhan City, Hubei Province of China.^1^ Despite only being reported to the World Health Organization (WHO) on December 31, 2019, COVID‐19 was declared a global health emergency on January 30, 2020.^2^ This was later classified as a Public Health Emergency of International Concern (PHEIC).^2^ The WHO subsequently declared COVID‐19 a global pandemic on March 11, 2020. This was the first such designation since the pandemic announcement of H1N1 influenza in 2009.^3^, ^4^

The impacts of COVID‐19 have been felt across the globe. However, even though the prevalence of COVID‐19 in Africa is relatively low, many believe that the impacts were felt more significantly in resource‐limited countries who already suffered from less established health‐care systems.^5^

As global health‐care networks offer their undivided attention toward the COVID‐19 pandemic, attention is drawn away from the far more established and equally devastating human immunodeficiency virus/acquired immune deficiency syndrome (HIV/AIDS) pandemic. Global efforts to fight COVID‐19 have resulted in a mismanagement of other illnesses such as HIV/AIDS.^5^ In addition to HIV/AIDS, some African countries were simultaneously facing other infectious diseases and viral outbreaks.^6^, ^7^ Consequently, many countries failed to hit the 2020 United Nations 90‐90‐90 treatment target to help end the AIDS epidemic within Africa.^8^, ^9^, ^10^ However, it is worth noting that HIV morbidity and mortality were not affected as severely as previously expected due to large disruptions to HIV management being less severe than anticipated.^11^, ^12^

The aggregation of two concurrent epidemics is known as a syndemic.^13^ This is when two or more epidemics interact synergistically to produce an increased burden of disease.^14^ Despite steadily improving efforts to address COVID‐19 within developing countries, previous efforts toward the treatment of pre‐existing infectious diseases such as HIV were currently inadequate.^10^, ^14^

Biochemical and lab‐based studies regarding COVID‐19 and HIV/AIDS show largely contradictory outcomes. Currently, it is suggested that immunosuppression associated with HIV/AIDS increases the risk of contracting COVID‐19 and results in more severe COVID‐19 symptoms.^15^, ^16^, ^17^ On the contrary, other studies indicate that a low CD4 cell count confers an advantage as it protects against a cytokine storm reaction.^18^, ^19^ It is therefore evident that this literature base is subject to much heterogeneity, and that further clarification is required before decisive clinical action can be taken.

The effects of COVID‐19 on the management of HIV/AIDS are of particular concern within African countries as these countries account for approximately two‐thirds of all HIV infections and deaths.^2^, ^20^ COVID‐19 has subsequently resulted in a reduced quality of care for HIV/AIDS patients globally.^5^, ^10^ However, this is of particular significance within Africa as in 2019, 17% of African HIV patients were undiagnosed and a further 30% went untreated.^21^ Consequently, COVID‐19 held the potential to further exacerbate HIV/AIDS care within Africa and to halt any progress that was being made.

More consideration must be given toward the interaction between COVID‐19 and HIV/AIDS. This attention must be paid in a pathophysiological, epidemiological, and geopolitical context. In an attempt to do achieve this, this review evaluates HIV/AIDS within Africa during COVID‐19 and outlines the current strategies attempting to minimize the negative impact of coronavirus on HIV care delivery. This review also aims to explore the current burden of these diseases and the future challenges that many African health‐care systems may face within this field.

## 2 HIV/AIDS IN AFRICA DURING COVID‐19

HIV has previously been referred to as a pandemic and according to 2019 data, 38.0 million people live with HIV (PLHIV) globally. Up to 25.7 million of these individuals (two‐thirds) originate from sub‐Saharan Africa, of which, around 20.7 million were from Eastern and Southern Africa and 4.9 million from Western and Central Africa.^22^ In addition, in 2019, the numbers of new HIV infections were 730,000 in Eastern and Southern Africa and 240,000 in Western and Central Africa. Consequentially, in 2019, the number of AIDS‐related deaths was 300,000 in Eastern and Southern Africa and 140,000 in a Western and Central Africa.^22^

Before the COVID‐19 pandemic, UNAIDS set their 90‐90‐90 target. This was an effort to ensure that 90% of PLHIV knew their disease status, 90% of people diagnosed with HIV were initiated on standard antiretroviral therapy (ART), and 90% of patients on ART had a suppressing viral load by 2020.^22^ Additionally, in 2015, the United Nations General Assembly introduced interim 2020 milestones in the 2016 Political Declaration on Ending AIDS which outlined their goals to reduce new annual HIV infections and AIDS‐related deaths to fewer than 500,0000 and to eliminate HIV‐related stigma and discrimination by 2020.^22^

Efforts to minimize the prevalence of HIV and its comorbidities were mostly successful and uninterrupted. Notably, in sub‐Saharan Africa, great strides had been taken in a bid to meet the UNAIDS 90‐90‐90 targets by the end of 2019. In Eastern and Southern Africa, 87% of PLHIV knew their status, 72% of PLWH were on treatment, and 65% of were virally suppressed by 2019. However, in Western and Central Africa, only 68% of PLWH knew their status, 58% of PLWH were on treatment, and 45% were virally suppressed.^22^ The figures indicated a slight but notable setback in achieving the UNAIDS targets by 2020.

Furthermore, it has been reported by the WHO that the number of people starting treatment in 2020 is far below what was expected.^18^ This was largely attributed to limited access to HIV/AIDS services such as HIV‐testing and treatment initiation. This in turn was correlated to the lockdown measures enforced in many parts of the world that prevented access to these vital health‐care services.^20^, ^23^

By the beginning of 2020, most African countries had integrated community‐based interventions into HIV health‐care service delivery to achieve the UNAIDS 90‐90‐90 targets. These interventions included follow‐up of patients on ART, reduction in transportation costs and the extension of HIV testing and treatment services to local health systems. However, these targets have still not been achieved by many African countries including Uganda, Zimbabwe, Malawi, Ethiopia, South Africa, Mozambique, and Nigeria.^24^ Only Rwanda and Botswana have made significant progress toward achieving the 90‐90‐90 target.^24^ Lockdown restrictions, despite successfully managing COVID‐19, were instrumental in preventing many of these countries reaching their 90‐90‐90 targets and further elucidate the negative implications of COVID‐19 on the care for PLHIV.^23^, ^25^

The already inadequate health‐care systems of many African countries were overwhelmed by the increased demands resulting from the COVID‐19 pandemic.^23^ This led to clinic closures, lack of pharmaceutical drugs, and loss of contact with many HIV patients.^23^, ^26^ Reallocating resources to aid the fight against coronavirus meant that many other essential health‐care services such HIV testing and treatment services suffered from inadequate resource allocation.^5^, ^10^ This led to a compromise in the quality of care received by PLHIV.^27^, ^28^ This in turn further slowed the progress toward attainment of the UNAIDS 90‐90‐90 targets.^24^

It is therefore essential that African governments take proactive measures to mitigate the disruptions in the supply of HIV/AIDS services irrespective of COVID‐19. Appropriate planning, financial allocation, and resource distribution will be fundamental in limiting the impacts of COVID‐19 on HIV/AIDS care delivery.

## 3 CURRENT EFFORTS

It is suggested that prioritizing COVID‐19 over HIV within Africa could lead to a 10‐year reversal of current HIV treatment progress.^10^, ^29^, ^30^ To prevent this, African leaders and organizations are currently undertaking several efforts to ensure rapid adaptation of HIV treatment programs in response to COVID‐19. These efforts can be broadly classified as follows.

### 3.1 Reorientation of HIV health‐care services

Many African countries have adopted multimonth (3–6 months) dispensing (MMD) of ART to PLHIV. This is opposed to a weekly regime as previously practiced. This has led to 50% reduction in the number of PLHIV clinic visits, thereby reducing COVID‐19 exposure.^31^ Furthermore, Burundi,^31^ Liberia,^32^ and other countries have scaled up HIV self‐testing and at‐home testing. This has helped to overcome stigma and structural barriers to HIV diagnosis.^33^ Finally, in Togo, Niger, and Namibia health‐care professionals have adopted the use of the telephone and social media for counseling and supporting PLHIV.^32^, ^33^

### 3.2 Strengthening community action

Nigeria, Burkina Faso, Mali, and many other countries have increased community engagement in the distribution of ART. Furthermore, in Namibia, community ART dispensing was expanded via the creation of new ART collection points in many communities via the use of mobile van and home delivery HIV services. An additional strategy being used in many African countries is the formation of community adherence groups that ensure PLHIV are compliant with ART and provide other support programs via virtual platforms.^33^

### 3.3 Creating a supportive environment for the continuous management of PLHIV

Before COVID‐19, Ghana has been successful in forming peer support groups to improve communication between PLHIV and to improve their access to care.^32^ However, with the implementation of travel restriction and social distancing, the provision of this support has been hindered. To adapt to this challenge, many African countries have switched to digital platforms to ensure continuity of the peer support program. Specific efforts cited in the virtual meeting on "The impact of the COVID‐19 on HIV programs in the ECOWAS region," organized by West‐Africa Health Organization, USAID and UNAIDS, include: the use of WhatsApp by trained health‐care workers to support counseling of people living with HIV in Togo; the opening of a hotline to communicate with PLHIV, tuberculosis patients, and key populations in Niger.^32^ Before COVID‐19 pandemic, the effectiveness of these digital tools in Africa had been illustrated by a study performed in Cameroon and Kenya.^34^ Within this study it was reported that two‐way text messaging supports for PLHIV markedly improved ART adherence and rates of viral suppression.^34^

### 3.4 Mitigating economic and food insecurity

Financial interventions by African governments have included a $1.4bn fiscal stimulus and grant to the poorest Nigerian populations as well as a $26bn economic package and new cash transfer scheme in South Africa. Other schemes include tax relief within Kenya.^20^, ^31^, ^32^, ^35^ It is well reported in Tanzania that food and cash incentive increased PLHIV retention in care and adherence to treatment.^36^

### 3.5 Addressing misinformation and misconception about HIV and COVID‐19

Many Africans (including PLHIV) have misconceptions about the origin of COVID‐19. Some believe it is a disease of a particular race and some believe the virus cannot survive in the tropics.^37^ In addressing this problem, many African countries are leveraging technologies to educate PLHIV and the public.^38^ For instance, the use of virtual senitization of key populations in Guinea Bissau and the collaboration of the Nigeria Centre for Disease Control with the United Nations Children's Fund to  launch a Short Message Service‐based  interactive  Chatbot to provide Nigerians with timely and accurate information on COVID‐19 were effective in raising awareness.^38^ The interplay between knowledge, attitude, and practice toward COVID‐19 among PLHIV was well documented in a correlational study in Rwanda.^39^ Within this study, a high prevalence of poor attitudes and misconceptions toward COVID‐19 was reported among the participants (26%). It was concluded that a good knowledge of COVID‐19 promoted positive attitudes toward the disease and in return the positive attitudes promoted good COVID‐19 practices which reduced the risk of PLHIV contracting the disease.^39^

In addition to these four key strategies, other notable efforts in Africa include cross‐border support which is well evidenced by Gambia loaning ART to Guinea‐Bissau to help solve supply interruption issues.^21^, ^32^ Despite COVID‐19's clear detrimental effects on HIV/AIDS healthcare within many African countries, it is encouraging to know that several authorities are undertaking proactive and effective management strategies to address the issue. However, as is made clear by the failing of many countries to meet their HIV health‐care targets, these efforts are not yet sufficient. These strategies must continue in a sustainable and financially viable manner if we are to see these countries reach their 90‐90‐90 targets.

## 4 FUTURE CHALLENGES

Despite the previously mentioned efforts to address these issues, by the end of 2019, 87% of PLHIV in Eastern and Southern Africa knew their disease status but only 72% were on treatment and only 65% of were virally suppressed. In Western and Central Africa, these numbers fall to 68%, 58%, and 45%, respectively.^22^ The emergence of COVID‐19 has therefore undoubtedly hindered Africa's progression toward achieving their 90‐90‐90 goals.^27^, ^28^

The aspects of HIV care that have been most greatly affected by COVID‐19 are those of HIV testing and ART treatment initiation.^10^, ^30^ This can be attributed to most of the African ART being manufactured outside the continent.^29^, ^38^ The closure of international borders has greatly affected the supply chain of ART and therefore poses a continual threat to the effective management of patients with HIV within Africa.

In addition, with the enforcement of lockdown and social distancing during the current pandemic, there has been a spike in human rights abuse, discrimination, and stigmatization of PLHIV.^40^ These factors have created further barriers within the accessibility of PLHIV to HIV services such as access to pre‐exposure prophylaxis and condoms. Therefore, it is not unreasonable to suggest that the hard‐won achievements within HIV care previously attained by many African countries could be somewhat disrupted by the COVID‐19 pandemic.^41^, ^42^ During COVID‐19 there has also been an increase in sexual violence due to the stay‐at‐home restrictions.^31^ Winnie Byanyima, the Executive Director of UNAIDS stated: “Sexual violence is a key driver of HIV infection, and the environment that makes a girl unsafe has been worsen by COVID‐19."^29^, ^38^

Finally, DW Africa, an African news corporation, reported that the lack of access to internet and high data cost within Africa has posed a challenge to the virtual smartphone support programs of health professionals, civil society organizations, community peer support group, and close relatives of PLHIV.^43^ COVID‐19 restrictions have undoubtedly affected many African health‐care systems and despite current efforts acting as positive first steps in mitigating these issues, the problem is far from solved. Sustainable and consistent ART supply, de‐stigmatization, sexual violence support, and access to technology are all additional strategies that must be addressed to ensure that HIV/AIDS patients can continue to receive the care that they require.

## 5 CONCLUSIONS AND FUTURE RECOMMENDATIONS

Within this area of research, it is unsurprising that COVID‐19 has exacerbated many of the already existing issues regarding HIV/AIDS care within Africa. These impacts may be considered in a biological, psychological, social, and political context. Consequentially, multimodal, and multidisciplinary approaches to these issues are those that are most likely to yield the most promising results.

Accessibility of HIV/AIDS care within Africa was limited before COVID‐19. However, access to care had improved in recent years and many countries were experiencing rapid health‐care reform. Nevertheless, the additional stresses that COVID‐19 placed on the African health‐care systems only served to worsen current issues and to hinder progress.

Before efforts can be made to continue improving the provision of both HIV/AIDS and COVID‐19 care in Africa, we have brought to the attention of the wider scientific and clinical communities that these two pathologies cannot be considered as separate entities. Rather, they must be considered as two potentially devastating diseases that are intrinsically linked through an array of biopsychosocial and geopolitical factors. It is now imperative to ensure that HIV/AIDS health‐care progress continues irrespective of coronavirus or any future barriers.

## CONFLICT OF INTERESTS

The authors declare that there are no conflict of interests.

## AUTHOR CONTRIBUTIONS

*Conceptualization, project administration, writing‐review and designing*: Olivier Uwishema. *Reviewed and edited the first draft*: Jeffrey Sun. R*eviewed and edited the second draft*: Helen Onyeaka. *Manuscript writing*: All authors. *Final approval of manuscript*: All authors.
